# Supplementary material for: Modeling glioblastoma heterogeneity as a dynamic network of cell states
Source: Mol Syst Biol. 2021 Sep 16;17(9):e10105. doi: 10.15252/msb.202010105 (PMC8444284; doi:10.15252/msb.202010105)
Supplement: Supplementary file 6 — Source Data for Figure 5 [file MSB-17-e10105-s004.zip › Figure5A_sourcedata/GSEA_3071/nosigdb_stateIV.GseaPreranked.1622795164626/SEURAT_CELLCYCLE.html]

Details for gene set SEURAT\_CELLCYCLE[GSEA]

|  || Dataset | state53071 |
| Phenotype | NoPhenotypeAvailable |
| Upregulated in class | na\_neg |
| GeneSet | SEURAT\_CELLCYCLE |
| Enrichment Score (ES) | -0.66888267 |
| Normalized Enrichment Score (NES) | -3.4227858 |
| Nominal p-value | 0.0 |
| FDR q-value | 0.0 |
| FWER p-Value | 0.0 |
Table: GSEA Results Summary

  

Fig 1: Enrichment plot: SEURAT\_CELLCYCLE      
 Profile of the Running ES Score & Positions of GeneSet Members on the Rank Ordered List

  

| PROBE | GENE SYMBOL | GENE\_TITLE | RANK IN GENE LIST | RANK METRIC SCORE | RUNNING ES | CORE ENRICHMENT || 1 | GAS2L3 |  |  | 244 | 1.161 | -0.0309 | No |
| 2 | CDCA7 |  |  | 818 | 0.596 | -0.1328 | No |
| 3 | PSRC1 |  |  | 1884 | 0.316 | -0.3331 | No |
| 4 | LBR |  |  | 2271 | 0.260 | -0.4037 | No |
| 5 | G2E3 |  |  | 2560 | -0.277 | -0.4552 | No |
| 6 | UBR7 |  |  | 2985 | -0.331 | -0.5322 | No |
| 7 | SMC4 |  |  | 3035 | -0.337 | -0.5369 | No |
| 8 | RAD51 |  |  | 3233 | -0.365 | -0.5698 | No |
| 9 | MCM2 |  |  | 3240 | -0.366 | -0.5659 | No |
| 10 | CHAF1B |  |  | 3451 | -0.400 | -0.6007 | No |
| 11 | TIPIN |  |  | 3559 | -0.420 | -0.6155 | No |
| 12 | CKAP2 |  |  | 3633 | -0.433 | -0.6236 | No |
| 13 | RANGAP1 |  |  | 3639 | -0.434 | -0.6185 | No |
| 14 | ECT2 |  |  | 3902 | -0.487 | -0.6622 | Yes |
| 15 | CDC25C |  |  | 3926 | -0.493 | -0.6598 | Yes |
| 16 | POLA1 |  |  | 3931 | -0.494 | -0.6538 | Yes |
| 17 | DSCC1 |  |  | 3962 | -0.501 | -0.6526 | Yes |
| 18 | SLBP |  |  | 3994 | -0.509 | -0.6516 | Yes |
| 19 | CENPF |  |  | 4003 | -0.511 | -0.6461 | Yes |
| 20 | NCAPD2 |  |  | 4055 | -0.526 | -0.6486 | Yes |
| 21 | WDR76 |  |  | 4060 | -0.527 | -0.6421 | Yes |
| 22 | NEK2 |  |  | 4086 | -0.536 | -0.6396 | Yes |
| 23 | EXO1 |  |  | 4094 | -0.539 | -0.6335 | Yes |
| 24 | BLM |  |  | 4100 | -0.541 | -0.6270 | Yes |
| 25 | CCNB2 |  |  | 4103 | -0.542 | -0.6199 | Yes |
| 26 | E2F8 |  |  | 4104 | -0.542 | -0.6125 | Yes |
| 27 | HMGB2 |  |  | 4243 | -0.578 | -0.6310 | Yes |
| 28 | BUB1 |  |  | 4246 | -0.578 | -0.6235 | Yes |
| 29 | MCM6 |  |  | 4273 | -0.590 | -0.6203 | Yes |
| 30 | BRIP1 |  |  | 4301 | -0.598 | -0.6173 | Yes |
| 31 | CCNE2 |  |  | 4564 | -0.714 | -0.6578 | Yes |
| 32 | TUBB4B |  |  | 4575 | -0.722 | -0.6498 | Yes |
| 33 | RFC2 |  |  | 4588 | -0.729 | -0.6421 | Yes |
| 34 | CKS2 |  |  | 4600 | -0.733 | -0.6341 | Yes |
| 35 | BIRC5 |  |  | 4618 | -0.745 | -0.6271 | Yes |
| 36 | RPA2 |  |  | 4638 | -0.760 | -0.6203 | Yes |
| 37 | TTK |  |  | 4644 | -0.762 | -0.6107 | Yes |
| 38 | USP1 |  |  | 4649 | -0.763 | -0.6010 | Yes |
| 39 | NUSAP1 |  |  | 4654 | -0.765 | -0.5912 | Yes |
| 40 | MSH2 |  |  | 4673 | -0.781 | -0.5839 | Yes |
| 41 | CENPA |  |  | 4683 | -0.788 | -0.5748 | Yes |
| 42 | CDC45 |  |  | 4702 | -0.798 | -0.5673 | Yes |
| 43 | GTSE1 |  |  | 4722 | -0.808 | -0.5598 | Yes |
| 44 | CDCA2 |  |  | 4732 | -0.816 | -0.5503 | Yes |
| 45 | KIF20B |  |  | 4745 | -0.825 | -0.5413 | Yes |
| 46 | CDCA8 |  |  | 4747 | -0.827 | -0.5301 | Yes |
| 47 | POLD3 |  |  | 4752 | -0.830 | -0.5194 | Yes |
| 48 | CENPE |  |  | 4757 | -0.832 | -0.5087 | Yes |
| 49 | KIF11 |  |  | 4760 | -0.835 | -0.4976 | Yes |
| 50 | CKAP2L |  |  | 4769 | -0.839 | -0.4876 | Yes |
| 51 | NUF2 |  |  | 4782 | -0.847 | -0.4782 | Yes |
| 52 | TMPO |  |  | 4790 | -0.851 | -0.4679 | Yes |
| 53 | MCM5 |  |  | 4792 | -0.853 | -0.4563 | Yes |
| 54 | PRIM1 |  |  | 4815 | -0.881 | -0.4484 | Yes |
| 55 | HELLS |  |  | 4819 | -0.882 | -0.4368 | Yes |
| 56 | KIF23 |  |  | 4825 | -0.891 | -0.4255 | Yes |
| 57 | MKI67 |  |  | 4836 | -0.902 | -0.4150 | Yes |
| 58 | DLGAP5 |  |  | 4857 | -0.922 | -0.4062 | Yes |
| 59 | UHRF1 |  |  | 4859 | -0.924 | -0.3937 | Yes |
| 60 | GMNN |  |  | 4873 | -0.939 | -0.3832 | Yes |
| 61 | CDCA3 |  |  | 4874 | -0.940 | -0.3703 | Yes |
| 62 | GINS2 |  |  | 4897 | -0.962 | -0.3613 | Yes |
| 63 | TACC3 |  |  | 4900 | -0.966 | -0.3483 | Yes |
| 64 | AURKA |  |  | 4906 | -0.973 | -0.3359 | Yes |
| 65 | MCM4 |  |  | 4947 | -1.011 | -0.3297 | Yes |
| 66 | DTL |  |  | 4970 | -1.042 | -0.3196 | Yes |
| 67 | ATAD2 |  |  | 4980 | -1.058 | -0.3067 | Yes |
| 68 | RRM1 |  |  | 4990 | -1.074 | -0.2937 | Yes |
| 69 | HMMR |  |  | 4993 | -1.080 | -0.2792 | Yes |
| 70 | FEN1 |  |  | 5014 | -1.113 | -0.2677 | Yes |
| 71 | AURKB |  |  | 5016 | -1.118 | -0.2525 | Yes |
| 72 | KIF2C |  |  | 5028 | -1.150 | -0.2388 | Yes |
| 73 | TOP2A |  |  | 5029 | -1.152 | -0.2229 | Yes |
| 74 | TPX2 |  |  | 5049 | -1.184 | -0.2102 | Yes |
| 75 | NDC80 |  |  | 5055 | -1.200 | -0.1947 | Yes |
| 76 | PCNA |  |  | 5060 | -1.214 | -0.1787 | Yes |
| 77 | CDC20 |  |  | 5082 | -1.249 | -0.1656 | Yes |
| 78 | UBE2C |  |  | 5106 | -1.314 | -0.1519 | Yes |
| 79 | CDC6 |  |  | 5107 | -1.314 | -0.1338 | Yes |
| 80 | CKS1B |  |  | 5121 | -1.338 | -0.1179 | Yes |
| 81 | HJURP |  |  | 5125 | -1.344 | -0.1000 | Yes |
| 82 | RAD51AP1 |  |  | 5133 | -1.375 | -0.0824 | Yes |
| 83 | TYMS |  |  | 5156 | -1.477 | -0.0663 | Yes |
| 84 | CDK1 |  |  | 5164 | -1.512 | -0.0468 | Yes |
| 85 | ANLN |  |  | 5183 | -1.577 | -0.0285 | Yes |
| 86 | CLSPN |  |  | 5202 | -1.663 | -0.0091 | Yes |
| 87 | RRM2 |  |  | 5232 | -1.875 | 0.0111 | Yes |
Table: GSEA details [plain text format]

  

Fig 2: SEURAT\_CELLCYCLE: Random ES distribution      
 Gene set null distribution of ES for **SEURAT\_CELLCYCLE**
